# Impact of type 2 diabetes mellitus in the utilization and in-hospital outcomes of surgical aortic valve replacement in Spain (2001–2015)

**DOI:** 10.1186/s12933-018-0780-2

**Published:** 2018-10-16

**Authors:** Ana López-de-Andrés, Napoleon Perez-Farinos, Javier de Miguel-Díez, Valentín Hernández-Barrera, Manuel Méndez-Bailón, José M. de Miguel-Yanes, Rodrigo Jiménez-García

**Affiliations:** 1 Preventive Medicine and Public Health Teaching and Research Unit, Health Sciences Faculty, Rey Juan Carlos University, Alcorcón, Madrid, Spain; 2 Department of Public Health and Psychiatry, Faculty of Medicine, Universidad de Malaga, Boulevard Louis Pasteur, 32, 28071 Málaga, Spain; 3 Respiratory Department, Hospital General Universitario Gregorio Marañón, Facultad de Medicina, Universidad Complutense de Madrid (UCM), Instituto de Investigación Sanitaria Gregorio Marañón (IiSGM), Madrid, Spain; 4 Internal Medicine Department, Hospital Universitario Clínico San Carlos, Facultad de Medicina, Universidad Complutense de Madrid (UCM), Madrid, Spain; 5 Internal Medicine Department, Hospital General, Universitario Gregorio Marañón, Madrid, Spain; 6 Facultad de Medicina, Universidad Complutense de Madrid (UCM), Madrid, Spain

**Keywords:** Type 2 diabetes mellitus, Surgical aortic valve replacement, Hospitalization, In-hospital mortality

## Abstract

**Background:**

The aims of this study were to examine trends in the incidence and in-hospital outcomes of SAVR among T2DM patients from 2001 to 2015, to compare clinical variables among T2DM patients and matched non-T2DM patients hospitalized for SAVR and to identify factors associated with in-hospital mortality (IHM) among T2DM patients.

**Methods:**

We performed a retrospective study using the Spanish National Hospital Discharge Database, 2001–2015. We included patients who had SAVR as the procedure in their discharge report. For each T2DM patient, we selected a sex-, age-, implanted valve type- and year-matched nondiabetic patient.

**Results:**

We identified 78,223 patients who underwent SAVR (23.49% with T2DM). The prevalence of T2DM increased significantly (p < 0.001) from 16.7% in 2001–2003 to 23.5% in 2012–2015. The incidence of SAVR increased significantly from 28.99 cases in 2001 to 65.79 cases in 2015 per 100,000 individuals in the T2DM population. Using Poisson regression models, we found that the incidence of SAVR was 2.60 times higher among patients with T2DM than among those without diabetes (IRR 2.60; 95% CI 2.56–2.65). The incidence of mechanical SAVR among T2DM patients remained stable from 2001 to 2015, and bioprosthetic SAVR rose from 8.29 to 41.74 cases per 100,000 individuals in the T2DM patient population (p < 0.001). We matched 8835 and 9543 patients who underwent mechanical and bioprosthetic SAVR, respectively. IHM decreased over time in T2DM patients and non-T2DM patients (from 8.89% and 7.81% to 3.88% and 5.07%, respectively). IHM was significantly lower in T2DM patients than in nondiabetic subjects who underwent bioprosthetic SAVR (4.77% vs. 6.04%, p < 0.001), with similar results obtained for mechanical valves (7.11% and 7.77%).

**Conclusions:**

The incidence of SAVR was higher in T2DM patients, and the incidence of bioprosthetic SAVR increased significantly among T2DM subjects. IHM decreased over time, regardless of the existence or absence of T2DM and the valve type. IHM was significantly lower in T2DM patients than in nondiabetic patients who underwent bioprosthetic SAVR.

## Background

For many decades, surgical aortic valve replacement (SAVR) was the recommended treatment for severe aortic valve stenosis [1]; mechanical or bioprosthetic valves have been the mainstream options [2], but these preferences have changed as transcatheter aortic valve replacement (TAVR) became the treatment of choice for patients with severe aortic stenosis, who are either inoperable or at high surgical risk [3]. Recently, Englum et al. [4] concluded that significant changes in the risk profiles of SAVR patients could be expected with the introduction of TAVR programs.

Diabetes mellitus adversely affects morbidity and mortality for major atherosclerosis-related cardiovascular diseases [5, 6]. Macro- and microvascular diseases are independently associated with the risk of major clinical microvascular events, major macrovascular events and death in patients with type 2 diabetes. The coexistence of these conditions is associated with the highest risks [7, 8].

In patients with aortic stenosis, diabetes was found to be second only to hypertension as the medical condition most associated with this stenosis [9]. Larsson et al. [10] reported that type 2 diabetes mellitus (T2DM) is independently associated with an increased risk of aortic valve stenosis (HR 1.34; 95% CI 1.05–1.71).

However, the mechanism initiating calcific aortic valve disease in diabetes is not well understood [11]. Mosch et al. compared inflammation and calcification using immunohistochemistry and immunofluorescence staining of calcific aortic valve disease patients with and without diabetes. These authors found that calcification and early calcification markers were significantly elevated in diabetic patients, concluding that diabetic patients could be molecularly in a more advanced disease stage with a higher grade of mineralization than nondiabetic patients [11].

Several studies have assessed the impact of diabetes on the outcomes of SAVR and concluded that T2DM is one of the predictors of poor outcomes after SAVR [12, 13]. Studies conducted in Spain and other countries have found that T2DM diabetic patients with aortic stenosis undergoing a valvular replacement procedure through SAVR or TAVR did not have higher mortality or complication rates than nondiabetic patients during hospitalization [14, 15].

In addition, the conflicting results of published studies led to the current research.

Using the SNHDD, we aim in this study to (i) examine trends in the incidence, characteristics and in-hospital outcomes of SAVR among patients with or without T2DM from 2001 to 2015; (ii) compare clinical variables in people with and without T2DM matched for implanted valve type, sex, age and year hospitalized for SAVR; and (iii) identify factors associated with IHM among patients with T2DM according to implanted valve type for SAVR.

## Methods

### Data source

This retrospective observational study was performed using the SNHDD. Details of the design and description of the SNHDD are available online. Briefly, this nationally representative database, which compiles all public hospital data, covers more than 95% of hospital admissions in Spain. The SNHDD includes patient variables (sex and date of birth), admission and discharge dates, up to 14 discharge diagnoses, and up to 20 procedures performed during the hospital stay [16].

### Patient population

We selected admissions of patients (aged ≥ 40 years) whose medical procedures included mechanical and bioprosthetic SAVR (ICD-9-CM codes: 35.21 and 35.22). Patients undergoing one or more additional cardiac procedures (defined as mitral, tricuspid or pulmonic valve replacement, repair or valvulotomy; replacement of the ascending aorta; closure of ventricular and atrial septal defects; ablation; and other rare procedures) were excluded. We collected data between January 1, 2001, and December 31, 2015.

We grouped admissions by diabetes status as follows: T2DM (ICD-9-CM codes 250.x0 and 250.x2) or no diabetes in any diagnostic position. We excluded people with type 1 diabetes mellitus (codes 250.x1 and 250.x3).

### Covariates

Clinical characteristics included information on overall comorbidity at the time of discharge, which was assessed by calculating the Charlson Comorbidity Index (CCI) [17]. Logically, the calculation of the CCI was performed by excluding diabetes as a disease.

Other diagnoses included in the CCI for analysis were chronic obstructive pulmonary disease (COPD) (ICD-9-CM codes 490, 491, 491.0, 491.1, 491.2x, 491.8, 491.9, 492, 492.0, 492.8, and 496), renal disease (ICD-9-CM codes 403.01, 403.11, 403.91, 404.02, 404.03, 404.12, 404.13, 404.92, 404.93, 582, 583.0–583.7, 585, 586, 588.0, V42.0, V45.1, and V56), coronary artery disease (ICD-9-CM codes 410–414), occlusive arterial disease (ICD-9-CM codes 0.93.0, 473.3, 440.x, 441.x, 443.1–443.9, 447.1, 557.1, 557.9, and V43.4) and atrial fibrillation (ICD-9-CM code 427.31).

Regardless of the position in the procedure coding list, we retrieved data on the following in-hospital procedures: coronary artery bypass graft (CABG) (ICD-9-CM codes 36.10–36.19) and pacemaker implantation (ICD-9-MC codes 37.70–37.74 and 37.80–37.83).

We evaluated the mean length of hospital stay (LOHS).

### Matching

In order to control the confounding effect of covariates and to assess the effect of T2DM on IHM and LOHS we tried to match each T2DM patients (n = 18,378) with a non-diabetic control. To do this we used the command CCMATCH of STATA 14.0. As matching variables we used; implanted valve type (mechanical or bioprosthetic), year of surgery, sex and year of birth. If more than one control was available for a case, the selection was conducted randomly. Doing this the program identified a non-diabetic control with identical age, sex, year of surgery and valves type for each diabetic patient. We could find 8814 non-diabetic controls for the 8835 diabetic patients who had undergone a mechanical SAVR (99.76%) and 9509 non-diabetic controls for the 9543 diabetic patients who had undergone a bioprosthetic SAVR (99.64%). As can be seen in Tables 2 and 3 the distribution of cases and control according to matching variables is identical. The analysis of the 55 diabetic patients that could not be matched shows that unmatched cases are significantly older (mean age 75.87 SD 17.01 vs. 71.87 SD 7.67; p < 0.001) and female in a higher proportion (58.18% vs. 42.12%; p = 0.044) than those that could be matched. However, beside the differences found, in our opinion, the very small proportion of cases that could not be matched (< 0.4%) is unlikely to affect our results.

### End points

The main end points in our investigation were trends in the incidence rates of hospitalization and IHM in patients whose medical procedure was mechanical and bioprosthetic SAVR. IHM was defined by the proportion of patients who died during admission for each year of study.

### Statistical analysis

To assess time trends, we estimated the incidence rates of admission for SAVR among T2DM and nondiabetic patients calculated per 100,000 individuals. We calculated T2DM-specific incidence rates by dividing the number of admissions per year, sex, and age group by the corresponding number of people in that population group using the age- and sex-adjusted estimated prevalence of T2DM obtained from National Health Surveys and based on data from the Di@bet.es Study, which estimated the prevalence of diabetes in the Spanish population [18, 19]. We also calculated incidence rates for nondiabetic patients by dividing the number of cases per year, sex, and age group by the corresponding number of people in that population group (excluding those with T2DM), according to the data from the Spanish National Institute of Statistics, as reported on 31 December of each year [20].

A descriptive statistical analysis was performed for all continuous variables and categories. Variables are expressed in proportions as means with standard deviations. A bivariable analysis according to year was performed using the χ^2^ test for linear trend (proportions) and ANOVA (means), as appropriate.

To assess differences between patients with and without T2DM, for each year and for the total sample, the statistical tests conducted for continuous variables were the T test for normal distributions and the Mann–Whitney test for non-normal distributions; categorical variables were compared using the Chi square test, and adjusted incidences were compared using Poisson regression. Estimates correspond to incidence rate ratios (IRR) with 95% confidence intervals (95% CI).

We constructed bivariable conditional logistic regression models to compare the study variables between patients with T2DM and matched controls. The analysis was stratified according to the type of SAVR.

To identify variables associated with IHM as a binary outcome among all patients with T2DM before matching, we performed three logistic regression analyses, one for each type of SAVR (mechanical, bioprosthetic and both types). The variables included in the multivariable models were those with significant results in the bivariable analysis and those considered relevant in other investigations. The estimates correspond to odds ratios (ORs) with 95% CI.

All statistical analyses were performed with Stata version 10.1 (Stata, College Station, Texas, USA). Statistical significance was set at p < 0.05 (2-tailed).

### Ethical aspects

The study maintained data confidentiality at all times. Given the anonymous and mandatory nature of the database, it was not necessary to obtain informed consent or approval from an ethics committee in accordance with Spanish legislation.

## Results

In our study, we identified a total of 78,223 hospitalizations of patients aged 40 years or more who underwent SAVR in Spain (2001–2015). Patients with T2DM accounted for 23.5% of the total population (10,629 men and 7749 women).

**Fig. 1:**
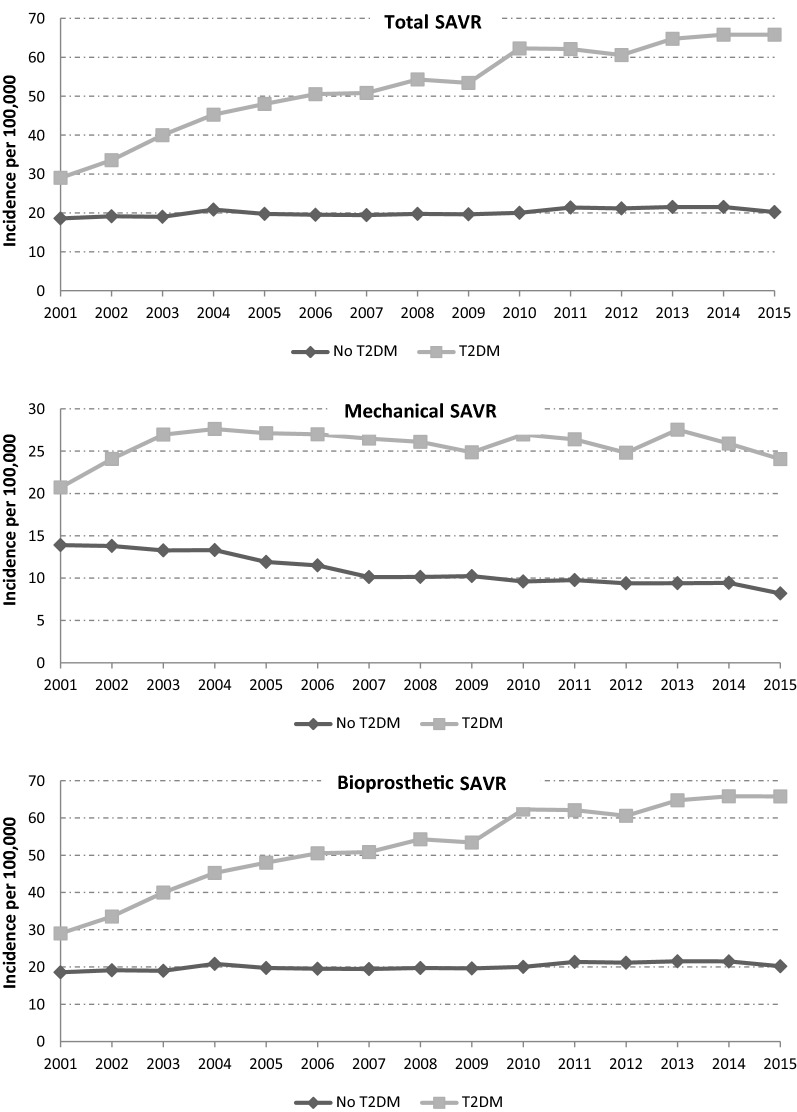
Trends of SAVR in T2MD and non-diabetic patients in Spain between 2001 and 2015 according to valve type

Figure 1 shows the trends of SAVR in T2DM and nondiabetic patients in Spain between 2001 and 2015 according to valve type.

Among patients with T2DM, we found that the incidence of SAVR coding increased significantly from 28.99 cases in 2001 to 65.79 in 2015 per 100,000 individuals in the T2DM population. In patients without T2DM, the incidence of admissions also increased significantly over the study period. The incidence was significantly higher in people with T2DM than in nondiabetic people for all years analyzed and for the type of valve implanted.

Using the Poisson regression model, adjusting for age and sex, we found that the incidence per population for SAVR was 2.60 times higher among patients with T2DM than among those without diabetes (IRR 2.60; 95% CI 2.56–2.65).

The incidence of mechanical SAVR among T2DM patients remained stable, with values oscillating between 20 and 28 cases per 100,000 individuals in the T2DM population from 2001 to 2015. The incidence of nondiabetic patients decreased significantly from 13.89 to 8.18 cases per 100,000 individuals in the non-T2DM population over the study period. The results of the Poisson regression models showed that the incidence per population for mechanical SAVR was 2.13 times higher among patients with T2DM than among those without T2DM (IRR 2.13; 95% CI 2.07–2.20).

Regarding bioprosthetic SAVR, the incidence was 8.29 and 4.68 in 2001 for patients with and without T2DM, respectively, and rose significantly to 41.74 and 12.01, respectively, in 2015. The incidence rate ratio (IRR) after adjusting for age and sex was 3.04 (95% CI 2.93–3.12).

Table 1 shows the clinical characteristics and in-hospital outcomes of patients with or without T2DM who underwent SAVR.

**Table 1:** Clinical characteristics and in-hospital outcomes of hospitalized patients who underwent surgical aortic valve replacement (SAVR) in Spain from 2001 to 2015 according to T2DM status

	T2DM	2001–2003	2004–2006	2007–2009	2010–2012	2013–2015	Total	Trend
Number of SAVR	Yes	1979	3001	3582	4637	5179	18,378	NA
	No	9897	11,329	11,832	12,955	13,832	59,845	NA
Prevalence of T2DM	Yes	16.7	20.9	23.2	26.4	27.2	23.5	< 0.001
Age, mean (SD)^abcdef^	Yes	70.2 (7.5)	70.9 (7.4)	71.6 (7.6)	72.5 (7.7)	72.8 (7.8)	71.9 (7.7)	< 0.001
	No	67.8 (9.5)	69.1 (9.6)	69.7 (9.8)	70.7 (10.1)	71.0 (10.0)	69.8 (9.9)	< 0.001
Female sex, n (%)^abcdf^	Yes	943 (47.6)	1312 (43.7)	1525 (42.6)	1934 (41.7)	2035 (39.3)	7749 (42.2)	< 0.001
	No	3795 (38.3)	4382 (38.7)	4582 (38.7)	5172 (39.9)	5398 (39.0)	23,329 (38.9)	0.079
CCI, mean (SD)^acef^	Yes	0.65 (0.5)	0.70 (0.6)	0.73 (0.7)	0.74 (0.6)	0.80 (0.7)	0.74 (0.7)	< 0.001
	No	0.59 (0.5)	0.67 (0.5)	0.69 (0.5)	0.73 (0.6)	0.76 (0.7)	0.70 (0.6)	< 0.001
Mechanical SAVR, n (%)^abcdef^	Yes	1384 (69.9)	1702 (56.7)	1747 (48.8)	1957 (42.2)	2045 (39.5)	8835 (48.1)	< 0.001
	No	7146 (72.2)	6909 (61.0)	6137 (51.9)	5952 (45.9)	5900 (42.6)	32,044 (53.5)	< 0.001
Bioprosthetic SAVR, n (%)^abcdef^	Yes	595 (30.1)	1299 (43.3)	1835 (51.2)	2680 (57.8)	3134 (60.5)	9543 (51.9)	< 0.001
	No	2751 (27.8)	4420 (39.0)	5695 (48.1)	7003 (54.1)	7932 (57.3)	27,801 (46.5)	< 0.001
CABG, n (%)^abcdef^	Yes	566 (28.6)	983 (32.8)	1133 (31.6)	1320 (28.5)	1432 (27.6)	5434 (29.6)	< 0.001
	No	1838 (18.6)	2348 (20.7)	2586 (21.9)	2745 (21.2)	2893 (20.9)	12,410 (20.8)	< 0.001
Pacemaker implantation, n (%)	Yes	77 (3.9)	121 (4.0)	189 (5.3)	193 (4.2)	234 (4.5)	814 (4.4)	0.419
	No	337 (3.4)	439 (3.9)	623 (5.3)	520 (4.0)	568 (4.1)	2487 (4.1)	0.030
LOHS, mean (SD)^abcef^	Yes	22.77 (16.6)	22.00 (21.1)	21.16 (18.0)	17.60 (13.7)	15.86 (12.9)	19.08 (16.4)	< 0.001
	No	20.41 (16.3)	19.89 (16.5)	19.48 (16.9)	17.64 (17.1)	16.36 (15.8)	18.59 (16.6)	< 0.001
IHM, n (%)^def^	Yes	176 (8.9)	230 (7.7)	254 (7.1)	226 (4.9)	201 (3.9)	1087 (5.9)	< 0.001
	No	773 (7.8)	895 (7.9)	833 (7.0)	799 (6.2)	701 (5.1)	4001 (6.7)	< 0.001

CABG, coronary artery bypass surgery; CCI, Charlson Comorbidity Index; LOHS, Length of hospital stay; IHM, in-hospital mortality, NA, not applicable

^a^p < 0.05 for difference when comparing patients with and without T2DM (2001–2003). ^b^ p < 0.05 for difference when comparing patients with and without T2DM (2004–2006). ^c^ p < 0.05 for difference when comparing patients with and without T2DM (2007–2009). ^d^ p < 0.05 for difference when comparing patients with and without T2DM (2010–2012). ^e^ p < 0.05 for difference when comparing patients with and without T2DM (2013–2015). ^f^ p < 0.05 for difference when comparing patients with and without T2DM (total)

The prevalence of T2DM among patients who underwent SAVR increased significantly (p < 0.001) from 16.7% in 2001–3 to 23.5% in 2012–2015.

In patients who underwent SAVR, there was a significant male predominance (57.84% for T2DM and 61.02% for no diabetes). Overall, patients with T2DM were older (71.88; SD = 7.71 years) than patients without diabetes (69.80; SD = 9.90 years) and had more coexisting medical conditions (mean CCI 0.74 ± 0.66 vs. 0.70 ± 0.63) (all p values < 0.05). Age and comorbidity increased significantly over time in both people with T2DM and those without diabetes. However, females were significantly more represented among patients with T2DM (47.65% in 2001–2003 vs. 39.29% in 2013–2015).

Over the entire period, T2DM patients were more likely to receive bioprosthetic valves than non-T2DM patients (51.93% vs. 46.46%; p < 0.05), whereas patients without diabetes were more likely to receive mechanical valves (53.54% vs. 48.07%; p < 0.05). The proportion of mechanical valves decreased significantly from 69.93% in 2001–2003 to 39.49% in 2013–2015 in patients with diabetes and from 72.2% in 2001–2003 to 42.65% in 2013–2015 in patients without T2DM. However, we detected a significant increase in bioprosthetic valves implanted in patients with and without diabetes (30.07% and 27.08%, respectively, in 2001–2003 vs. 60.51% and 57.35% in 2013–2015).

Overall, T2DM patients who received SAVR required concomitant CABG more frequently than non-T2DM patients (29.57% vs. 20.74%). In patients without diabetes, the use of CABG increased significantly during the study period; however, in T2DM patients, we found a reduction over time (28.6% in 2001–2003 vs. 27.65% in 2013–2015; p < 0.001).

The use of pacemaker implantation increased significantly in non-T2DM patients (3.41% in 2001–2003 vs. 4.11% in 2013–2015). No differences were found between the two groups of patients.

The overall mean LOHS was significantly higher in patients with T2DM (19.08 vs. 18.59 days). Over time, the LOHS decreased significantly in both patients with and without diabetes.

For the total time period, crude IHM was 5.91% for T2DM patients and 6.69% for nondiabetic individuals (p < 0.05). IHM decreased significantly over time in both patients with and without T2DM (from 8.89% and 7.81%, in 2001–2003 to 3.88% and 5.07% in 2013–2015, respectively) (Table 1).

Tables 2 and 3 show the distribution and IHM according to the study variables of T2DM patients and matched nondiabetic controls who underwent mechanical (Table 2) and bioprosthetic valve replacement (Table 3).

**Table 2:** Distribution and in-hospital mortality according to the study variables of type 2 diabetes (T2DM) patients and matched nondiabetic controls with a mechanical surgical aortic valve replacement (SAVR)

	T2DM	Matched non-T2DM	P	IHM T2DM	IHM matched non-T2DM	p
Year, n (%)						
2001–2003	1380 (15.7)	1380 (15.7)	NA	140 (10.1)	116 (8.4)	0.116
2004–2006	1696 (19.2)	1696 (19.2)		134 (7.9)	148 (8.7)	0.382
2007–2009	1747 (19.8)	1747 (19.8)		143 (8.2)	152 (8.7)	0.585
2010–2012	1954 (22.2)	1954 (22.2)		115 (5.9)	143 (7.3)	0.073
2013–2015	2037 (23.1)	2037 (23.1)		95 (4.7)	126 (6.2)	0.031
Sex, n (%)						
Male	5260 (59.7)	5260 (59.7)	NA	317 (6.0)	387 (7.4)	0.006
Female	3554 (40.3)	3554 (40.3)		310 (8.7)	298 (8.4)	0.611
Age in years, mean (SD)	68.65 (8.2)	68.65 (8.2)	NA	71.3 (7.6)	71.56 (7.6)	0.732
Age group, mean (SD), years						
40–64	2624 (29.8)	2624 (29.8)	NA	114 (4.3)	119 (4.5)	0.740
65–74	3837 (43.5)	3837 (43.5)		277 (7.2)	287 (7.5)	0.662
75–84	2275 (25.8)	2275 (25.8)		227 (9.9)	272 (11.9)	0.033
≥ 85	78 (0.9)	78 (0.9)		9 (11.5)	7 (8.9)	0.566
CCI, mean (SD)	0.7 (0.7)	0.7 (0.6)	0.033	1.2 (0.9)	1.13 (0.9)	0.742
CCI, n (%)						
0	4286 (48.6)	4306 (48.8)	0.061	159 (3.7)	199 (4.6)	0.133
1	2982 (33.8)	3127 (35.5)		261 (8.7)	265 (8.5)	0.810
2+	1546 (17.5)	1381 (15.7)		207 (13.4)	221 (16)	0.593
COPD, n (%)						
No	7903 (89.7)	8049 (91.3)	< 0.001	559 (7.1)	621 (7.7)	0.073
Yes	911 (10.3)	765 (8.7)		68 (7.5)	64 (8.4)	0.495
Renal dysfunction, n (%)						
No	8075 (91.6)	8287 (94.0)	< 0.001	533 (6.6)	618 (7.5)	0.046
Yes	739 (8.4)	527 (6.0)		94 (12.7)	67 (12.7)	0.410
Coronary artery disease, n (%)						
No	5133 (58.2)	6255 (71.0)	< 0.001	301 (5.9)	401 (6.4)	0.226
Yes	3681 (41.8)	2559 (29.0)		326 (8.9)	284 (11.1)	0.201
Occlusive peripheral arterial disease, n (%)						
No	7815 (88.7)	7236 (82.1)	< 0.001	553 (7.1)	561 (7.7)	0.216
Yes	999 (11.3)	1578 (17.9)		74 (7.4)	124 (7.9)	0.866
Atrial fibrillation, n (%)						
No	6224 (70.6)	6034 (68.5)	0.002	432 (6.9)	489 (8.1)	0.080
Yes	2590 (29.4)	2780 (31.5)		195 (7.5)	196 (7.0)	0.222
CABG, n (%)						
No	6341 (71.9)	7128 (80.9)	< 0.001	420 (6.6)	480 (6.7)	0.636
Yes	2473 (28.1)	1686 (19.1)		207 (8.4)	205 (12.2)	0.118
Pacemaker implantation, n (%)						
No	8466 (96.0)	8446 (95.8)	0.442	609 (7.2)	656 (7.8)	0.106
Yes	348 (3.9)	368 (4.2)		18 (5.2)	29 (7.9)	0.146
LOHS, mean (SD)	20.1 (17.7)	19.1 (17.2)	< 0.001	24.7 (23.6)	25.01 (24.4)	0.617

The p value for the difference between patients with type 2 diabetes and matched controls was calculated with the bivariate conditional logistic regression model

CABG, Coronary artery bypass surgery; CCI, Charlson Comorbidity Index; COPD, chronic obstructive pulmonary disease; LOHS, length of hospital stay; IHM, in-hospital mortality; NA, not applicable, as this is a matching variable

**Table 3:** Distribution and in-hospital mortality according to the study variables of type 2 diabetes (T2DM) patients and matched nondiabetic controls with a bioprosthetic surgical aortic valve replacement (SAVR)

	T2DM	Matched non-T2DM	p	IHM T2DM	IHM Matched non-T2DM	p
Year, n (%)						
2001–2003	588 (6.2)	588 (6.2)	NA	35 (5.9)	39 (6.6)	0.638
2004–2006	1288 (13.5)	1288 (13.5)		94 (7.3)	104 (8.1)	0.459
2007–2009	1831 (19.3)	1831 (19.3)		111 (6.1)	128 (6.9)	0.253
2010–2012	2676 (28.1)	2676 (28.1)		110 (4.1)	141 (5.3)	0.045
2013–2015	3126 (32.9)	3126 (32.9)		104 (3.3)	163 (5.2)	< 0.001
Sex, n (%)						
Male	5346 (56.2)	5346 (56.2)	NA	243 (4.5)	288 (5.4)	0.045
Female	4163 (43.8)	4163 (43.8)		211 (5.1)	287 (6.9)	< 0.001
Age in years, mean (SD)	74.86 (5.7)	74.86 (5.7)	NA	75.6 (5.4)	75.9 (5.7)	0.845
Age group, mean (SD), years						
40–64	398 (4.2)	398 (4.2)	NA	16 (4.0)	19 (4.8)	0.591
65–74	3757 (39.5)	3757 (39.5)		158 (4.2)	179 (4.8)	0.247
75–84	5150 (54.2)	5150 (54.2)		267 (5.2)	359 (6.9)	< 0.001
≥ 85	204 (2.1)	204 (2.1)		13 (6.4)	18 (8.8)	0.339
CCI, mean (SD)	0.7 (0.7)	0.7 (0.6)	0.011	1.23 (1)	1.14 (0.9)	0.469
CCI, n (%)						
0	4600 (48.4)	4681 (49.2)	0.046	118 (2.6)	156 (3.3)	0.195
1	3235 (34.0)	3274 (34.4)		172 (5.3)	232 (7.1)	0.186
2+	1674 (17.6)	1554 (16.3)		164 (9.8)	187 (12.0)	0.006
COPD, n (%)						
No	8597 (90.4)	8584 (90.3)	0.754	403 (4.7)	523 (6.1)	< 0.001
Yes	912 (9.6)	925 (9.7)		51 (5.6)	52 (5.6)	0.067
Renal dysfunction, n (%)						
No	8383 (88.2)	8731 (91.8)	< 0.001	370 (4.4)	499 (5.7)	< 0.001
Yes	1126 (11.8)	778 (8.2)		84 (7.5)	76 (9.8)	0.782
Coronary artery disease, n (%)						
No	5237 (55.1)	6240 (65.6)	< 0.001	194 (3.7)	320 (5.1)	< 0.001
Yes	4272 (44.9)	3269 (34.4)		260 (6.1)	255 (7.8)	0.599
Occlusive peripheral arterial disease, n (%)						
No	8564 (90.1)	8271 (86.9)	< 0.001	404 (4.7)	503 (6.1)	< 0.001
Yes	945 (9.9)	1238 (13.0)		50 (5.3)	72 (5.8)	0.178
Atrial fibrillation, n (%)						
No	6385 (67.1)	5968 (62.8)	< 0.001	316 (4.9)	375 (6.3)	0.380
Yes	3124 (32.8)	3541 (37.2)		138 (4.4)	200 (5.6)	0.013
CABG, n (%)						
No	6553 (68.9)	7200 (75.7)	< 0.001	285 (4.3)	384 (5.3)	< 0.001
Yes	2956 (31.1)	2309 (24.3)		169 (5.7)	191 (8.3)	0.184
Pacemaker implantation, n (%)						
No	9044 (95.1)	9078 (95.5)	0.248	430 (4.7)	549 (6.0)	< 0.001
Yes	465 (4.9)	431 (4.5)		24 (5.2)	26 (6.0)	0.215
LOHS, mean (SD)	18.2 (14.9)	18.2 (17.1)	0.706	24.6 (18.5)	28.0 (21.0)	0.569

The p value for the difference between patients with type 2 diabetes and matched controls was calculated with the bivariate conditional logistic regression model

CABG, coronary artery bypass surgery; CCI, Charlson Comorbidity Index; COPD, chronic obstructive pulmonary disease; LOHS, length of hospital stay; IHM, in-hospital mortality; NA, not applicable, as this is a matching variable

Patients with T2DM who underwent mechanical SAVR had significantly more comorbidity (mean CCI, 0.73 ± 0.66 vs. 0.71 ± 0.62, p = 0.033) and a higher prevalence of COPD (10.34% vs. 8.68%, p < 0.001), renal dysfunction (8.38% vs. 5.98%, p < 0.001) and coronary artery disease (41.76% vs. 29.03%, p < 0.001) than the control nondiabetic patients. However, patients with diabetes had a lower prevalence of occlusive peripheral arterial disease and atrial fibrillation (Table 2).

The use of concomitant CABG was higher in T2DM patients than in matched non-T2DM patients (28.06% vs. 19.13%, p < 0.001). The mean LOHS was higher in patients with diabetes (20.08 days vs. 19.1 days; p < 0.001).

No differences were found for IHM between patients with T2DM and matched non-T2DM controls who underwent mechanical valve procedures, with rates of 7.11% and 7.77%, respectively.

Patients with T2DM who underwent bioprosthetic SAVR had higher values of CCI (0.74 ± 0.67 vs. 0.71 ± 0.64, p = 0.011) and a higher prevalence of renal dysfunction (11.84% vs. 8.18%, p < 0.001) and coronary artery disease (44.93% vs. 34.38%, p < 0.001) than non-T2DM controls. However, T2DM patients had a lower prevalence of occlusive peripheral arterial disease and atrial fibrillation (9.94% and 32.85%, respectively vs. 13.02% and 37.24%, all p < 0.001). The use of concomitant CABG was higher in T2DM patients than in matched non-T2DM patients (31.09% vs. 24.28%, p < 0.001).

As shown in Table 3, we found that IHM was higher in matched nondiabetic patients (6.04%) than in patients with T2DM (4.77%) (p < 0.001).

When we compared T2DM patients who underwent mechanical SAVR with patients with T2DM who underwent bioprosthetic SAVR, we found that the first group of patients were younger (68.65 years vs. 74.86 years; p < 0.001) and more likely to have coronary artery disease (44.93% vs 41.76%; p < 0.05) and to require concomitant CABG (31.09% vs 28.06%; p < 0.05). T2DM patients who received mechanical valves had a longer LOHS (20.08 days vs. 18.16 days; p < 0.05) and a higher IHM (7.11% vs. 4.77%; p < 0.05) than T2DM patients who received bioprosthetic valves.

Table 4 shows the results of the logistic regression analysis to identify the factors independently associated with IHM in T2DM patients according to the type of SAVR.

**Table 4:** Multivariable analysis of factors associated with in-hospital mortality among type 2 diabetes (T2DM) patients according to the type of surgical aortic valve replacement (SAVR)

	Mechanical SAVR OR (95% CI)	Bioprosthetic SAVR OR (95% CI)	Both types of SAVR OR (95% CI)
Years			
2001–2003	1	1	1
2004–2006	0.75 (0.58–0.96)	1.14 (0.76–1.70)	0.85 (0.69–1.05)
2007–2009	0.78 (0.61–1.00)	0.90 (0.61–1.33)	0.80 (0.65–0.98)
2010–2012	0.51 (0.39–0.67)	0.60 (0.40–0.89)	0.52 (0.42–0.65)
2013–2015	0.39 (0.30–0.52)	0.47 (0.32–0.70)	0.41 (0.33–0.50)
Sex			
Female	1.47 (1.24–1.74)	1.29 (1.06–1.57)	1.39 (1.23–1.59)
Age groups (years)			
40–64	1	1	1
65–74	1.56 (1.24–1.97)	0.96 (0.58–1.59)	1.49 (1.22–1.84)
75–84	2.21 (1.74–2.82)	1.23 (0.75–2.02)	2.01 (1.63–2.49)
≥ 85	2.83 (1.44–5.56)	1.83 (0.89–3.77)	2.82 (1.79–4.43)
CCI			
0	1	1	1
1	2.62 (2.13–3.22)	2.22 (1.74–2.82)	2.44 (2.09–2.85)
2+	4.50 (3.61–5.61)	4.45 (3.47–5.70)	4.50 (3.82–5.31)
CABG	1.27 (1.06–1.52)	1.30 (1.06–1.59)	1.28 (1.12–1.47)
SAVR type			
Mechanical	NA	NA	1.66 (1.45–1.90)

Only those variables with a significant association are shown

CABG, coronary artery bypass surgery; CCI, Charlson Comorbidity Index; OR, odds ratio obtained using logistic regression models; 95% CI: 95% confidence intervals; NA, not applicable

Among T2DM patients who underwent mechanical SAVR, IHM was significantly higher in women (OR 1.47; 95% CI 1.24–1.74), older subjects (OR 2.83, 95% CI 1.44–5.56 for ≥ 85 years old vs. < 40–64 years old), individuals with more comorbidities according to the CCI (vs. no comorbidities, OR 4.50, 95% CI 3.61–5.61 for ≥ 2 comorbidities) and patients with concomitant CABG (OR 1.27, 95% CI 1.06–1.52).

As shown in Table 4, female sex (OR 1.29, 95% CI 1.06–1.57), concomitant CABG use (OR 1.30, 95% CI 1.06–1.59) and comorbidities increase the risk of IHM in patients with T2DM with bioprosthetic SAVR.

Finally, after multivariable adjustment, mechanical valves SAVR was associated with a significantly higher IHM (OR, 1.66; 95% CI 1.45–1.90) among T2DM patients than among those who underwent bioprosthetic SAVR in our study.

## Discussion

The main result of our investigation is the great increase in the number of T2DM patients who underwent SAVR in Spain from 2001 to 2015.

In Spain, the prevalence of T2DM among patients undergoing SAVR increased from 16.7% to 23.5% over the study period (2001–2015). Brown et al. described changes in the isolated aortic valve replacement population over 10 years in the Society of Thoracic Surgeons National Database [21]. These authors found that the prevalence of diabetes rose by 64.6% from 15.5% in 1997 to 25.4% in 2006 [21]. Additionally, from 2009 to 2015 in the USA, among Medicare beneficiaries with SAVR, the prevalence of diabetes increased from 19.7% to 31.6% [22]. In the single-center study conducted by Silaschi et al. the prevalence of diabetes was 13.8% in 2002 and reached 17.7% in 2012 among SAVR patients, with the total number of surgeries increasing from 139 to 322 [23]. The results of the Virginia Cardiac Services Quality Initiative database showed that the prevalence of diabetes in 2002–2008 was 30.0%, 33.0% in 2009–2011 and 36.7% in 2012–2015 (p < 0.01) [24]. The expected aging of the Spanish population will surely result in an increase in the prevalence of T2DM patients among those undergoing SAVR in the next decade [14].

The global increase in SAVR incidence is consistent with the trend observed in other European countries [25, 26]. A large study based on national registry data in the Netherlands showed that SAVR was more than twice as high in 1995 compared with 2010 [26]. The authors concluded that this trend can partly be attributed to an increased prevalence of valvular heart disease and an increasing proportion of diseased patients diagnosed as such. Because the mean age of the patients has risen, both factors are likely to have played an important role [25, 26].

Studies from the USA between 2003 and 2015 using the Nationwide Inpatient Sample and data on Medicare beneficiaries showed that the utilization trends of SAVR rose constantly [22, 27]. Culler et al. described that the number of Medicare beneficiaries undergoing SAVR with tissue or mechanical valves grew at a 3.1% compounded annual growth rate from 2009 to 2015 [22].

We found that the incidence rates of hospitalization of SAVR in patients with T2DM were higher than those in patients without T2DM. Furthermore, the use of SAVR has doubled among T2DM patients. This finding could be due to several factors, such as advanced age and a high index of comorbidities, leading to an increased risk of hospitalization for T2DM and SAVR [12]. In addition, improvements in treatment in terms of short-term and long-term complications have broadened the indication for surgery over the years [28].

Brennan et al. suggested that there is a chance that the increase in overall aortic valve replacement volume is the result of increased diagnosis and consequent referral of high-risk patients with symptomatic aortic valve stenosis, yet it is possible that the introduction of TAVR led to increases in the treatment of lower risk patients or, alternatively, very high-risk patients who may or may not derive therapeutic benefit [29]. Silaschi et al. agreed that the introduction of TAVR may have led to an increased overall caseload of procedures performed on the aortic valve, suggesting a high-level recruitment phenomenon [23].

Several studies have analyzed the effect of TAVR introduction on the use of SAVR, with contradictory results [4, 22, 23, 29, 30]. As mentioned before, in the United States, the overall SAVR volumes seem to have risen modestly since the approval of TAVR [4, 22, 29]. In Europe, where TAVR was started years earlier, the number of SAVRs has remained stable or slightly declined, while TAVR utilization has increased constantly since its introduction [22, 30].

The introduction of TAVR seems to have affected the number of SAVRs in Spain among T2DM patients [14]. As shown in Fig. 1, since 2013, the incidence of this procedure has remained stable, with rates of 64.75 (n = 1726), 65.81 (n = 1746) and 65.79 (n = 1707) cases per 100,000 individuals in the T2DM populations for 2013, 2014 and 2015, respectively. The future use of SAVR among diabetic patients will be influenced by the clinical results of TAVR when patients with moderate surgical risk undergo this technology.

Thus far, the outcomes with TAVR in T2DM patients are conflicting in published reports [31–33]. In a recent sub-analysis of the Placement of Aortic Transcatheter Valves clinical trial, mortality after 1 year follow-up was higher in non-T2DM individuals [31]. However, a German study found that T2DM patients undergoing TAVR had a worse prognosis with higher short- and long-term mortality [32].

In Israel, 443 patients (35.6% suffering diabetes) with severe aortic stenosis receiving TAVR were followed for two years, and the study revealed that diabetes was not associated with increased mortality [33].

As we expected, a substantial reduction in the rate of implanted mechanical valves was observed, and we found an increase in the use of bioprosthetic valves. A study using the National Inpatient Sample (NIS) found an increase in the use of bioprosthetic valves from 37.7% in 1998–2001 to 63.6% in 2007–2011. These authors found that patients with diabetes received more bioprosthetic valves than mechanic valves (23.7% vs. 21.0%), possibly because of their higher age [34]. This trend has also been reported in other studies conducted in Europe and the USA [23, 25–27, 35], and these results suggest improved durability of biological prostheses, fewer neurological and functional complications and avoidance of permanent anticoagulation [23, 36].

Lastly, technological advances such as the valve-in-valve transcatheter procedure have provided new alternatives to reoperations in biological prostheses [26].

As in the general population, T2DM patients with coronary artery disease, atrial fibrillation and renal failure are more likely to receive bioprosthetic than mechanical valves [34]. Age plays a greater role in bioprosthetic valve selection for patients with comorbidities than for those without, with a notably greater role for coronary artery disease patients requiring revascularization [34]. We observed that valve choice in T2DM is influenced by age, with most patients aged > 74 years receiving bioprosthetic valves and patients with a mean age of 68.65 years receiving mechanical valves.

We found that the IHM of all types of SAVR has decreased significantly over the last 15 years in both patients with T2DM and those without T2DM. Siregar et al. [26] found that IHM for SAVR with or without CABG decreased significantly from 3.5% in 2007 to 2.4% in 2010. A similar trend was found for operative mortality in most other studies and databases, which could reflect a combination of improved health care in general, more healthy aging and gradual improvements in cardiac surgery over time [21, 22, 25].

We propose that in our country, the introduction of TAVR may have had a beneficial influence on the mortality rate of SAVR by subjecting more patients with high-risk T2DM to TAVR instead of SAVR. In a recent report from the Spanish National Society of Cardiology evaluating heart interventions from 2010 to 2015, 73.2% of patients undergoing TAVR were not elected for SAVR and were at very high surgical risk [37].

Our results show that T2DM patients have lower (bioprosthetic) or similar (mechanical) mortality after SAVR than do nondiabetic patients. Halkos et al. [12] found that diabetes was not a predictor of IHM (OR, 0.86; 95% CI, 0.49–1.50). The lower IHM in T2DM patients undergoing SAVR compared to that in nondiabetic patients might be multifactorial. Obesity is more prevalent in T2DM patients undergoing SAVR, and this effect might have contributed to the decrease in IHM previously mentioned [14, 38].

In our study, aortic valve replacement in T2DM patients with a bioprosthetic valve, compared to those with a mechanical valve, was associated with lower IHM, which is consistent with observational evidence [34, 39]. In the general population, bioprosthetic valves are associated with lower IHM than mechanical prostheses are, which come at the cost of slightly higher rates of in-hospital complications [34]. Du et al. [39] examined 66,453 Medicare beneficiaries aged > 65 years who underwent SAVR between 2006 and 2011 and found that the risk of death on the date of surgery was 60% higher for mechanical-valve recipients than for bioprosthetic-valve recipients. Isaacs et al. [34] found higher IHM among patients who received mechanical valves (5.2%) than among those with bioprosthetic valves (4.4%).

Female sex and more comorbidities are factors associated with IHM in patients with T2DM. In agreement with these findings, a study using NIS data from 166,809 patients who underwent SAVR between 2003 and 2014 found that IHM was significantly higher in women than in men (5.6% versus 4%) [27]. The onset mechanism for cardiovascular disease, the delayed presentation of valve problems and/or the later referral of women to cardio-thoracic surgery may explain some of the differences in risk profile [40]. This worse result after SAVR among women calls for urgent investigations to identify and reduce these significant differences.

Diabetes is a predictor of long-term mortality for patients having SAVR and CABG [41]. In our study, we found significantly higher IHM in T2DM patients with concomitant CABG, independent of the type of valve used, than in T2DM patients without this procedure.

There are some points that should be taken into consideration when interpreting the results of the present study. Our data source was the SNHDD, an administrative database that contains discharge data for hospitalizations in Spain and uses information that the physician has included in the discharge report [16]. Coding practices, as well as errors in coding, may differ between individual physicians and institutions. Thus, our results are subject to several potential biases, including differences in the capture of adverse outcomes across hospitals or even diabetes diagnosis during the study period.

Our findings are limited by the lack of data on some relevant clinical parameters, such as glycosylated hemoglobin measurement, which did not have blood glucose levels to evaluate the degree of control of diabetes during admissions, treatments during hospitalization or left ventricular ejection fraction. The absence of these parameters may affect the analysis and limit the generalizability of this study. We also lack information on diabetes duration, which has been associated with major adverse cardiovascular events in the presence of arterial disease [42].

Despite these limitations, the quality and validity of our dataset have been assessed and determined to be useful for health research [43].

## Conclusions

In conclusion, our study reveals that the incidence of SAVR was higher in T2DM patients than in those without this disease and that it increases over time in both groups of patients. In both patient groups, mechanical SAVR decreased and the use of bioprosthetic valves increased over time. IHM decreased over time regardless of the existence or absence of T2DM, despite a concomitant increase in SAVR procedures during the same period. IHM was significantly lower in T2DM patients who underwent bioprosthetic SAVR. However, no differences were found in T2DM patients who underwent SAVR with mechanical valves. Higher mortality rates in T2DM patients were associated with female sex, the presence of comorbidities, increasing age (except in bioprosthetic valves) and concomitant CABG. Remarkably, IHM was higher among T2DM patients who underwent mechanical SAVR than among those who underwent bioprosthetic valves. However, given the methodological limitations of administrative data, more prospective investigations aimed at evaluating the influence of SAVR in T2DM patients with aortic stenosis are needed.

## Abbreviations

CABG: coronary artery bypass graft
CCI: Charlson Comorbidity Index
COPD: chronic obstructive pulmonary disease
ICD-9-CM: International Classification of Diseases, Ninth Revision, Clinical Modification
IHM: in-hospital mortality
LOHS: length of hospital stay
NIS: National Inpatient Sample
SAVR: surgical aortic valve replacement
SNHDD: Spanish National Hospital Discharge Database
T2DM: type 2 diabetes mellitus
TAVR: transcatheter aortic valve replacement

## References

[1] Carabello BA, Paulus WJ (2009). Aortic stenosis. Lancet.

[2] Suri RM, Schaff HV (2013). Selection of aortic valve prostheses: contemporary reappraisal of mechanical versus biologic valve substitutes. Circulation.

[3] Nishimura RA, Otto CM, Bonow RO, Carabello BA, Erwin JP, Fleisher LA (2014). 2014 AHA/ACC guideline for the management of patients with valvular heart disease: a report of the American College of Cardiology/American Heart Association Task Force on Practice Guidelines. J Thorac Cardiovasc Surg.

[4] Englum BR, Ganapathi AM, Schechter MA, Harrison JK, Glower DD, Hughes GC (2016). Changes in risk profile and outcomes of patients undergoing surgical aortic valve replacement from the pre- to post-transcatheter aortic valve replacement eras. Ann Thorac Surg.

[5] Dinesh Shah A, Langenberg C, Rapsomaniki E, Denaxas S, Pujades-Rodriguez M, Gale CP (2015). Type 2 diabetes and incidence of a wide range of cardiovascular diseases: a cohort study in 1·9 million people. Lancet.

[6] de Miguel-Yanes JM, Jiménez-García R, Hernández-Barrera V, Méndez-Bailón M, de Miguel-Díez J, Lopez-de-Andrés A (2017). Impact of type 2 diabetes mellitus on in-hospital-mortality after major cardiovascular events in Spain (2002–2014). Cardiovasc Diabetol.

[7] Mohammedi K, Woodward M, Marre M, Colagiuri S, Cooper M, Harrap S (2017). Comparative effects of microvascular and macrovascular disease on the risk of major outcomes in patients with type 2 diabetes. Cardiovasc Diabetol..

[8] Mohammedi K, Woodward M, Hirakawa Y, Zoungas S, Colagiuri S, Hamet P (2016). Cardiovasc Diabetol..

[9] Yan AT, Koh M, Chan KK, Guo H, Alter DA, Austin PC (2017). Association between cardiovascular risk factors and aortic stenosis: the CANHEART Aortic Stenosis Study. J Am Coll Cardiol.

[10] Larsson SC, Wallin A, Håkansson N, Stackelberg O, Bäck M, Wolk A (2018). Type 1 and type 2 diabetes mellitus and incidence of seven cardiovascular diseases. Int J Cardiol.

[11] Mosch J, Gleissner CA, Body S, Aikawa E (2010). Histopathological assessment of calcification and inflammation of calcific aortic valves from patients with and without diabetes mellitus. Histol Histopathol..

[12] Halkos ME, Kilgo P, Lattouf OM, Puskas JD, Cooper WA, Guyton RA (2010). The effect of diabetes mellitus on in-hospital and long-term outcomes after heart valve operations. Ann Thorac Surg.

[13] Smith RL, Herbert MA, Dewey TM, Brinkman WT, Prince SL, Ryan WH (2012). Does body mass index affect outcomes for aortic valve replacement surgery for aortic stenosis?. Ann Thorac Surg.

[14] Mendez-Bailon M, Lorenzo-Villalba N, Muñoz-Rivas N, de Miguel-Yanes JM, De Miguel-Diez J, Comín-Colet J (2017). Transcatheter aortic valve implantation and surgical aortic valve replacement among hospitalized patients with and without type 2 diabetes mellitus in Spain (2014–2015). Cardiovasc Diabetol.

[15] Abramowitz Y, Jilaihawi H, Chakravarty T, Mangat G, Maeno Y, Kazuno Y, Takahashi N, Kawamori H, Cheng W, Makkar RR (2016). Impact of diabetes mellitus on outcomes after transcatheter aortic valve implantation. Am J Cardiol.

[16] Instituto Nacional de Gestión Sanitaria, Ministerio de Sanidad, Servicios Sociales e Igualdad. Conjunto Mínimo Básico de Datos, Hospitales del INSALUD. http://www.ingesa.msssi.gob.es/estadEstudios/documPublica/CMBD-2001.htm. Accessed 16 June 2018.

[17] Charlson ME, Pompei P, Ales KL, MacKenzie CR (1987). A new method of classifying prognostic comorbidity in longitudinal studies: development and validation. J Chronic Dis.

[18] Ministerio de Sanidad, Servicios Sociales e Igualdad. [Encuesta Nacional de Salud de España]. http://www.msssi.gob.es/estadEstudios/estadisticas/encuestaNacional/. Accessed 16 June 2018.

[19] Soriguer F, Goday A, Bosch-Comas A, Bordiú E, Calle-Pascual A, Carmena R (2012). Prevalence of diabetes mellitus and impaired glucose regulation in Spain: the Di@betes Study. Diabetologia..

[20] . Instituto Nacional de Estadística. Population estimates. 2010. http://www.ine.es. Accessed 16 June 2018.

[21] Brown JM, O’Brien SM, Wu C, Sikora JA, Griffith BP, Gammie JS (2009). Isolated aortic valve replacement in North America comprising 108,687 patients in 10 years: changes in risks, valve types, and outcomes in the Society of Thoracic Surgeons National Database. J Thorac Cardiovasc Surg.

[22] Culler SD, Cohen DJ, Brown PP, Kugelmass AD, Reynolds MR, Ambrose K (2018). Trends in aortic valve replacement procedures between 2009 and 2015: has transcatheter aortic valve replacement made a difference?. Ann Thorac Surg.

[23] Silaschi M, Conradi L, Treede H, Reiter B, Schaefer U, Blankenberg S (2016). Trends in surgical aortic valve replacement in more than 3,000 consecutive cases in the era of transcatheter aortic valve implantations. Thorac Cardiovasc Surg.

[24] Hawkins RB, Downs EA, Johnston LE, Mehaffey JH, Fonner CE, Ghanta RK (2017). Impact of transcatheter technology on surgical aortic valve replacement volume, outcomes, and cost. Ann Thorac Surg.

[25] Dunning J, Gao H, Chambers J, Moat N, Murphy G, Pagano D (2011). Aortic valve surgery: marked increases in volume and significant decreases in mechanical valve use—an analysis of 41,227 patients over 5 years from the Society for Cardiothoracic Surgery in Great Britain and Ireland National database. J Thorac Cardiovasc Surg.

[26] Siregar S, de Heer F, Groenwold RH, Versteegh MI, Bekkers JA, Brinkman ES (2014). Trends and outcomes of valve surgery: 16-year results of Netherlands Cardiac Surgery National Database. Eur J Cardiothorac Surg.

[27] Chaker Z, Badhwar V, Alqahtani F, Aljohani S, Zack CJ, Holmes DR (2017). Sex differences in the utilization and outcomes of surgical aortic valve replacement for severe aortic stenosis. J Am Heart Assoc.

[28] Baumgartner H, Falk V, Bax JJ, De Bonis M, Hamm C, Holm PJ (2018). 2017 ESC/EACTS guidelines for the management of valvular heart disease. Rev Esp Cardiol (Engl Ed)..

[29] Brennan JM, Holmes DR, Sherwood MW, Edwards FH, Carroll JD, Grover FL (2014). The association of transcatheter aortic valve replacement availability and hospital aortic valve replacement volume and mortality in the United States. Ann Thorac Surg.

[30] Gaede L, Kim WK, Blumenstein J, Liebetrau C, Dörr O, Nef H (2017). Temporal trends in transcatheter and surgical aortic valve replacement: an analysis of aortic valve replacements in Germany during 2012–2014. Herz..

[31] Lindman BR, Pibarot P, Arnold SV, Suri RM, McAndrew TC, Maniar HS (2014). Transcatheter versus surgical aortic valve replacement in patients with diabetes and severe aortic stenosis at high risk for surgery: an analysis of the PARTNER Trial (Placement of Aortic Transcatheter Valve). J Am Coll Cardiol.

[32] Puls M, Bleckmann A, Jacobshagen C, Danner BC, Hasenfuß G, Seipelt R, Schillinger W (2014). Diabetes increases short- and long-term mortality after transcatheter aortic valve implantation (TAVI). Dtsch Med Wochenschr.

[33] Berkovitch A, Segev A, Barbash I, Grossman Y, Maor E, Erez A (2015). Clinical impact of diabetes mellitus in patients undergoing transcatheter aortic valve replacement. Cardiovasc Diabetol..

[34] Isaacs AJ, Shuhaiber J, Salemi A, Isom OW, Sedrakyan A (2015). National trends in utilization and in-hospital outcomes of mechanical versus bioprosthetic aortic valve replacements. J Thorac Cardiovasc Surg.

[35] Fujita B, Ensminger S, Bauer T, Möllmann H, Beckmann A, Bekeredjian R (2018). Trends in practice and outcomes from 2011 to 2015 for surgical aortic valve replacement: an update from the German Aortic Valve Registry on 42 776 patients. Eur J Cardiothorac Surg.

[36] Chan V, Jamieson WR, Germann E, Chan F, Miyagishima RT, Burr LH (2006). Performance of bioprostheses and mechanical prostheses assessed by composites of valve-related complications to 15 years after aortic valve replacement. J Thorac Cardiovasc Surg.

[37] García Del Blanco B, Jiménez Quevedo P, Diaz JD, Hernández F, Rumoroso JR, Sabaté M (2017). Spain: coronary and structural heart interventions from 2010 to 2015. EuroIntervention.

[38] Lavie CJ, McAuley PA, Church TS, Milani RV, Blair SN (2014). Obesity and cardiovascular diseases: implications regarding fitness, fatness, and severity in the obesity paradox. J Am Coll Cardiol.

[39] Du D, McKean S, Kelman JA, Laschinger J, Johnson C, Warnock R (2014). Early mortality after aortic valve replacement with mechanical prosthetic vs bioprosthetic valves among Medicare beneficiaries: a population-based cohort study. JAMA Intern Med.

[40] Ibrahim MF, Paparella D, Ivanov J, Buchanan MR, Brister SJ (2003). Gender-related differences in morbidity and mortality during combined valve and coronary surgery. J Thorac Cardiovasc Surg.

[41] Akins CW, Hilgenberg AD, Vlahakes GJ, MacGillivray TE, Torchiana DF, Madsen JC (2002). Results of bioprosthetic versus mechanical aortic valve replacement performed with concomitant coronary artery bypass grafting. Ann Thorac Surg.

[42] Noh M, Kwon H, Jung CH, Kwon SU, Kim MS, Lee WJ, Park JY, Han Y, Kim H, Kwon TW, Cho YP (2017). Cardiovasc Diabetol..

[43] Ribera A, Marsal JR, Ferreira-González I, Cascant P, Pons JM, Mitjavila F (2008). Predicting in-hospital mortality with coronary bypass surgery using hospital discharge data: comparison with a prospective observational study. Rev Esp Cardiol.

